# The Treatment of Uterine Fibroids

**Published:** 1900-02-03

**Authors:** 


### THE TREATMENT OF UTERINE FIBROIDS.

At the same meeting Dr. Gow read a paper on the operative treatment of uterine fibroids, his remarks being based on a series of 47 cases which he had removed by abdominal hysterectomy, with intra-peritoneal treatment of the stump. The indications for the operation were, he said, largely bound up with the mortality from it. So long as this remained as high as 10 per cent., or even less, the operation must necessarily be restricted to cases where life was seriously threatened; whereas, if the mortality were low, the operation might fairly be advised where the symptoms were sufficiently severe to cause invalidism. Among the 47 instances which he recorded, without selection, of abdominal hysterectomy for fibroids, where the stump was treated intra-peritoneally, there was only one death, which was due to secondary hæmorrhage 36 hours after the operation. In four of his cases a pyosalpinx was present on one or both sides, but no evil consequences followed. In only one of these did he employ drainage. He thought that the essentials to success were control over bleeding vessels and complete asepsis, and with a view to the latter he urged simplification of abdominal operations. One assistant was all that was necessary, and six sponges and six pairs of pressure forceps were usually amply sufficient. The paper supported the view that of all the radical operations for fibroids the one referred to was the best, and that it might be expected in the future to give a mortality of not more than 1 or 2 per cent.

